# Design Principles as a Guide for Constraint Based and Dynamic Modeling: Towards an Integrative Workflow

**DOI:** 10.3390/metabo5040601

**Published:** 2015-10-16

**Authors:** Christiana Sehr, Andreas Kremling, Alberto Marin-Sanguino

**Affiliations:** Specialty Division for Systems Biotechnology, Technische Universität München, Boltzmannstraße 15, Garching 85748, Germany; E-Mails: christiana.sehr@tum.de (C.S.); a.kremling@lrz.tu-muenchen.de (A.K.)

**Keywords:** systems biology, FBA, BST, thermodynamic feasibility analysis

## Abstract

During the last 10 years, systems biology has matured from a fuzzy concept combining omics, mathematical modeling and computers into a scientific field on its own right. In spite of its incredible potential, the multilevel complexity of its objects of study makes it very difficult to establish a reliable connection between data and models. The great number of degrees of freedom often results in situations, where many different models can explain/fit all available datasets. This has resulted in a shift of paradigm from the initially dominant, maybe naive, idea of inferring the system out of a number of datasets to the application of different techniques that reduce the degrees of freedom before any data set is analyzed. There is a wide variety of techniques available, each of them can contribute a piece of the puzzle and include different kinds of experimental information. But the challenge that remains is their meaningful integration. Here we show some theoretical results that enable some of the main modeling approaches to be applied sequentially in a complementary manner, and how this workflow can benefit from evolutionary reasoning to keep the complexity of the problem in check. As a proof of concept, we show how the synergies between these modeling techniques can provide insight into some well studied problems: Ammonia assimilation in bacteria and an unbranched linear pathway with end-product inhibition.

## 1. Introduction

The complexity of high-throughput datasets has reached a level, where mathematical models are needed to understand biochemical networks. The most common way of modeling metabolic networks, if regulation is to be included, is a system of differential equations describing the dynamics of certain variables, normally metabolite concentrations. These state variables are collected as a vector of dependent variables $x_{d}\left(t\right)$. Unlike the dependent variables, whose dynamics are determined by the system itself, there are variables whose dynamics are determined by external processes or clamped at a constant value, these variables will be collected in the vector of independent variables $x_{i}\left(t\right)$. The connectivity of the network is represented by the stoichiometric matrix S and can be determined by collecting a list of the active reactions in the cell and their respective stoichiometries. Such a list can be obtained from a genome and refined by experiments and literature searches as is explained in many texts about metabolic reconstructions [1]. The equations of the system would be:

(1) $${\dot{x}}_{d}=S\;v\left(x_{d}\left(t\right),x_{i}\left(t\right)\right)$$

The function of reaction rates $v(x_{d}\left(t\right),x_{i}\left(t\right))$ presents a problem that is difficult to solve. These functions represent complicated kinetics that involve undetermined parameters. Unlike the different components that can be grouped as variables of the system, which can be measured by a wide variety of techniques (proteomics, transcriptomics, metabolomics), there are no equivalent omics techniques able to measure kinetic parameters on a large scale. Even worse, the more variables we measure, the more kinetic parameters will be needed! This often frustrates attempts to reverse engineer biological systems by measuring their dynamics and using them to determine the parameters of the model. The alternative approach for which we will advocate in this work is to constrain the possible values of the components to eliminate any parameter combination that is not feasible, then concentrate on identifying likely evolutionary strategies and possible ways to achieve them. We can reduce the amount of states to consider and therefore make a much better use of the information we get by discarding anything that is impossible and focusing our attention on evolutionary sensible alternatives.

### The Chess Metaphor

If we were walking among the tables during a chess championship without having any knowledge about the game, we might wonder why some moves are recurrent and some are never seen or why some pieces tend to be in certain areas of the board. Most games start with the same movements, advancing one of the central pawns, and no game ever starts with some pawn at the side. Moreover, the pawns are never seen behind their starting positions and the bishops have a tendency to show up along the main diagonals. Some of these questions can be easily explained from the rules themselves. Pawns can only move forwards and that is why they cannot reach any position behind their starting point. But nothing in the rules prevent the player from starting the game with the leftmost pawn, so why doesn’t that ever happen? In order to understand this, we would have to think in terms of winning and losing strategies. Moving the central pawns satisfies two important goals: controlling the center of the board and clearing the way for other pieces. Domination of the center can also be achieved by moving a knight towards it, and that is also an opening move that is sometimes seen. Advancing the leftmost pawn, however legal a move, does not follow any strategy with chances of winning and it is therefore not done. Chess masters do not think in terms of isolated moves but in sets of concerted moves that pursue certain goals. Independent moves can be much better understood within the context of these strategies, which are sometimes complementary but can also be conflicting: should I make an offensive move or surrender the initiative to defend my king by “castling”?

Just as the rules of chess constrain the possible moves in a game, physics and chemistry constrain all the possible phenotypes available to evolution. Moreover, just as the most frequent moves in a game are those that enable successful strategies, the most frequent genotypes are those that enable its own propagation. Finally, just as the chess games of our example only covered a fraction of all possible combinations, so can metabolic networks, as products of evolution, be expected to concentrate in those “designs” that enable a certain performance. This prevalence of successful strategies has been observed in many biological systems. The analysis by Shoval *et al.* [2] shows this using a few examples, one of them, Darwin’s finches. Let’s classify finches according to two parameters: body size and beak shape. These two parameters define a whole space of possible “designs” and each species of finch will occupy a point in it. Certain designs can be considered optimal for a certain task, in this case, different diets can benefit from different beak morphologies and body sizes: Long beaks and medium body sizes are more appropriate for birds on a diet of insects or nectar, large beaks and a large bodies are favored for hard seed eaters and, finally, small body sizes and small beaks are more appropriate for eating small soft seeds. Each of these three phenotypes, optimally adapted to follow a single strategy, has been called archetype, and the distance of a given phenotype to each of them will reflect how successful this phenotype will be when following the corresponding strategy. Taking these three archetypes as vertices of a triangle, any phenotype within the triangle will be a trade-off between strategies. Moving in any direction will improve the performance in some strategies at the cost of hampering the others. This space is called the Pareto front and can be defined as a sort of optimality, since leaving the Pareto front means moving away from all strategies and closer to none. By plotting the position of many types of finches in this space, Shoval *et al.* showed that phenotypes tend to be in the Pareto space and large areas of perfectly possible phenotypes are never found in nature.

**Figure 1:**
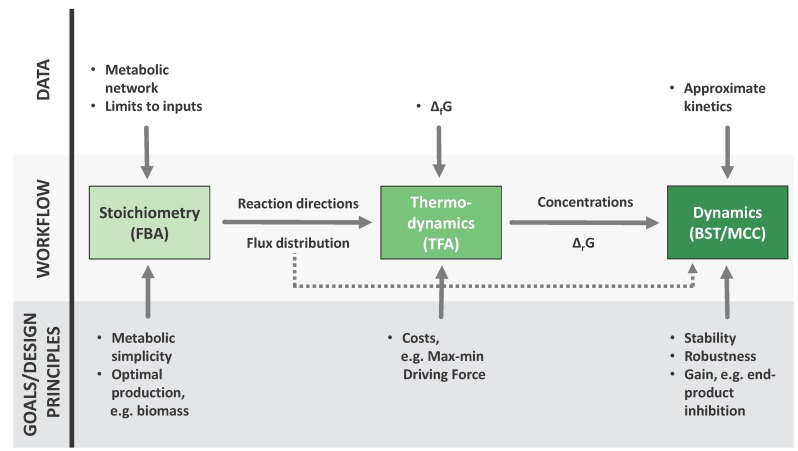
A sequential workflow from metabolic reconstructions to dynamic models. Each of the three techniques featured here incorporates different kinds of information into the process, this information can be of two kinds: different types of data, shown in the upper part of the figure, and evolutionary considerations at different levels, shown in the lower part of the figure. Furthermore, the results of each step are fed to the next. The flux distributions provided by FBA (Flux Balance Analysis) [4,5] can be used as an input to TFA (Thermodynamic Feasibility Analysis) [6] and the results of both approaches can be translated to parameters for a dynamic model, which can be formulated and analyzed accoding to BST (Biochemical Systems Theory) [7,8,9] and MCC (mathematically controlled comparison) [47].

In this work, we will try to show how the sequential application of several well established strategies (see Figure 1) for the analysis of biological systems provides a systematic workflow towards understanding the “design principles” that result in successful metabolic networks. The term “design principle” has been present since the stone age of systems biology [3], and can be seen as analogous to a software design pattern: A reusable solution to a commonly occurring problem in a given—here evolutionary—context. By analyzing a given network from a flux oriented perspective (Flux Balance Analysis (FBA)) [4,5], then refining the analysis through thermodynamic analysis and finally using the resulting information to constrain the definition of a dynamic model, many impossible alternatives will be discarded. Moreover, identifying the pros and cons of the different alternative models goes beyond reducing the amount of expected phenotypes, like in the example of Darwin’s finches; it will also organize the probable outcomes in meaningful evolutionary terms.

## 2. Established Methods

### 2.1. Flux Centric Approaches: Constraining the Flux Space

In growing microbes, fitness can be equated to growth rate. It is therefore reasonable to start considerations on evolutionary strategies for microbes by inspecting potential for growth of a given metabolic network.

In the steady state, since ${\dot{x}}_{d}=0$, the dynamic Equation (1) that describes the system gets simplified to a linear system of algebraic equations and the fluxes of the system can be taken as the only variables. Such a system admits infinitely many solutions, but each solution is, in principle, a valid flux distribution according to the principle of mass conservation. The most popular way of exploring the solution space is precisely to maximize the biomass production flux using linear programming.

Let’s define the production of biomass, ${\text{v}}_{\mathrm{bio}}$, as one of the rates of the system, collected in rate vector v. Then, the maximal biomass production will be the solution of:

$$\begin{array}{cc} \text{max}\; & {\text{v}}_{\mathrm{bio}} \end{array}$$

$$\begin{array}{cc} \text{s.t:}\; & \end{array}$$

$$\begin{array}{cc} & S\;v=0 \end{array}$$

(2) $$\begin{array}{cc} & v_{L}\leq v\leq v_{U} \end{array}$$

where $v_{L}$ and $v_{U}$ are lower and upper bounds established for the fluxes. In order to avoid the trivial solution ${\text{v}}_{\mathrm{bio}}=\infty$, an upper limit has to be imposed to the uptake of some limiting nutrient: ${\text{v}}_{\mathrm{in}}\leq {\text{v}}_{\text{in}}^{\text{max}}$. This limit can be imposed on different kinds of input, like carbon source or oxygen.

One approach to this problem is to establish an arbitrary upper limit for ${\text{v}}_{\mathrm{in}}$—let’s say 100. Since all the values are conditioned by this arbitrary upper bound, each flux ${\text{v}}_{k}$ by itself has no meaningful units and the solution must be interpreted as a vector of yields: $Y_{\text{k,in}}=\frac{v_{\text{k}}}{{\text{v}}_{\mathrm{in}}}$. There has often been confusion based on this distinction between optimal rates and optimal yields. As has been discussed in detail elsewhere [10], these two magnitudes are not only different, they may even be conflicting goals—see Crabtree effect [11] or overflow metabolism.

Another popular aproach is to establish a reasonable limit for the uptake rate of the carbon source. This may however obscure the meaning of the solution, since it implies that the limiting factor for growth is limited uptake rate. Even if the result of this optimization matches the data, it can lead to a misinterpretation of the results. To help clarify this point, we will reformulate the problem in an equivalent version. Swapping the objective function and one of the constraints yields the following problem:

$$\begin{array}{cc} \text{min}\; & v_{\text{in}} \end{array}$$

$$\begin{array}{cc} \text{s.t:}\; & \end{array}$$

$$\begin{array}{cc} & S\;v=0 \end{array}$$

(3) $$\begin{array}{cc} & v_{L}\leq v\leq v_{U} \end{array}$$

Here, the former limiting factor, $v_{\text{in}}$, is left unbound and used as a minimization goal. Now it is the former objective function, $v_{\text{bio}}$, which is limited by some element external to the model—e.g., an upper limit on the number of ribosomes per cell—so it cannot exceed a certain value, ${\text{v}}_{\text{bio}}^{\text{max}}$. The problem is then completed by imposing that the system fulfills the condition: $v_{\text{bio}}\geq {\text{v}}_{\text{bio}}^{\text{max}}$, to guarantee that the solution stays at maximum growth. This reformulation of a linear problem [12] is often used in trade-off analysis and it is a well established method in multi-criteria programming [13]. Since the additional constraint on $v_{\text{bio}}$ forces the solution to produce biomass at the maximum rate, the whole feasible area of the second problem includes the optimal solution of the first, and since ${\text{v}}_{\mathrm{in}}$ reaches its upper limit in the first problem, its minimum value compatible with Equation (3) defines the solution of Equation (2). A detailed proof of the equivalence of the two formulations is beyond the scope of this work, but the reader can find it in texts on multi-criteria optimization like [13]. We will instead focus in showing how these alternative formulations of the problem can explain the same datasets and yet lead to completely different conclusions.

**Figure 2:**
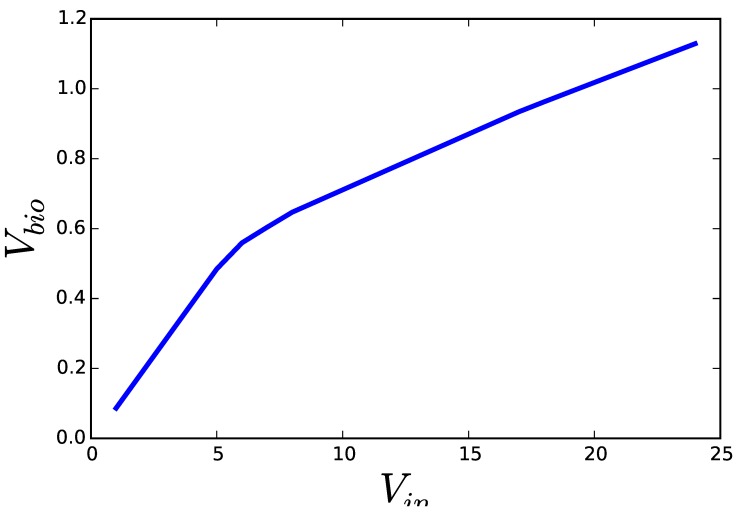
Results of applying the two optimization programs discussed in the text on a genome scale model of *E. coli.* Solving Equation (2) for increasing upper limits for glucose uptake ($V_{in}$) yields solutions ascending along the blue curve. Solving Equation (3) for decreasing lower limits on the growth rate ($V_{bio}$) results in solutions descending along the same curve. All optimizations were performed using COBRA for python version 0.3.2 and the E. coli model iJO1366 included with the software. All fluxes were optimized with their default limits except EX_o2_e, for which a lower bound of −10.0 was set.

Figure 2 shows the result of solving both formulations of a FBA problem (growth of *E. coli* on glucose under limited oxygen availability). While the solutions to Problem (2) should be interpreted as the flux distribution that achieves the fastest growth, when the cell incorporates glucose as fast as it can, the same solution obtained from Problem (3) must be interpreted as the flux distribution that is able to achieve a pre-established growth rate, while making the most efficient use of glucose. In the first case, overcoming the single bottleneck—e.g., overexpression of the glucose transporter—would be enough to increase growth and all the other fluxes. In this second case, the flux distribution, including glucose uptake, is the result of a complex set of regulatory mechanisms that adapt the metabolic fluxes to an already pre-established growth rate. A cell operating the assumptions of the second formulation would not grow faster, if the single glucose transporter was over-expressed. Furthermore, once the metabolic fluxes satisfy the need for growth, the cell could take some additional substrate to produce side products that inhibit the growth of competing organisms. This is known to happen with overflow metabolism. Moreover, side products accumulated in the medium can be metabolized after the primary nutrient is exhausted, leading to a second phase of growth.

In the next section, we will summarize how Thermodynamic Feasibility Analysis (TFA) is performed and show with an example how it constrains but also complements FBA.

### 2.2. Thermodynamics: The Bridge to Metabolites

Thermodynamic descriptions of a system at equilibrium are built upon pairs of conjugate variables, one intensive (x) and one extensive (y), so that the product xdy has units of energy or work—e.g., pressure (P) and volume (V). Thus, the contribution of each pair to a thermodynamic potential is the product of a certain driving force (x) and the displacement or flow (y) it provokes, like in PdV. This force/flow relationship is also present in non-equilibrium thermodynamics, so any system admits many different representations depending on the chosen combination of flows and forces. e.g., entropy is often replaced with temperature through a Legendre transform. Models of biochemical processes have also been shown to be amenable to such alternative representations both in the linear [14] and non-linear domain [15]. In theory, any constraint, to which the system is subjected, can be written in terms of the forces (chemical affinities/metabolite concentrations) or the flows (metabolic fluxes), but in practice, the information on many constraints is only available as a function of one set of variables or the other. Starting with an under-constrained system, like FBA, based on only the flow representation, can already be extremely useful to generate possible states for the system. Now, thermodynamics can be used to filter out unrealistic distributions. A first round of thermodynamic tests is already state of the art when performing FBA. Thermodynamically unfeasible cycles are removed through different techniques like minimal norm solutions [16], loopless FBA [17] or enumeration of rays and linealities [18].

But thermodynamics goes beyond filtering solutions. The framework, called Thermodynamic Feasibility Analysis [6,19,20], enables the complementation of flux-centric descriptions of the system, providing information about the conjugate variables (thermodynamic forces/metabolite concentrations). Since fluxes are already constrained in the formulation of FBA, the next step is to find constraints for the admissible metabolite concentrations. Metabolite levels are constrained from above (solubility limit and ionic strength) and from below (they cannot be much smaller than the observed lower limit for $K_{M}$ [21] or the enzymes acting on them would not be able to carry any flux). Standard boundaries based on previous observations can be chosen, and they tend to be between 0.1 μM and 10 mM, except for some special cases, such as gases or phosphate [20,21,22]. The chosen boundaries for the metabolites can then be propagated to their chemical potentials, since the definition of Gibbs free energy involves both the energies and the concentrations.

Feasible states are defined as a set of linear inequalities, which can be explored using the same tools that make FBA possible:

(4) $$\begin{array}{c} g\;=\;g^{0}+R\;T\;S^{T}\;y\;\;\leq \;\;0 \end{array}$$

where the vector g contains the ΔG for each reaction, $g^{0}$ the standard free energies and y is a vector containing the logarithms of the concentrations following the convention $y_{i}=\ln x_{i}$. An immediate way of summarizing the space defined by Equation (4) is using linear programming to calculate the minimum and maximum admissible levels for each metabolite as well as the maximum and minimum possible values for ΔG in each reaction.

**Figure 3:**
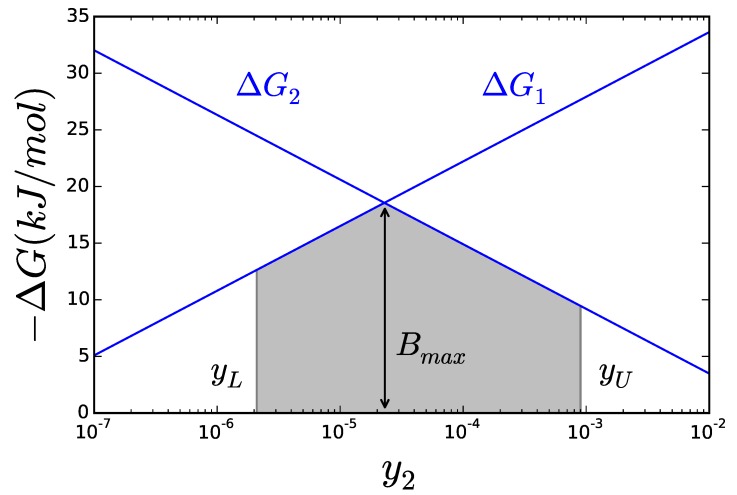
The lines represent the ΔG of each reaction. The area below both curves (shaded) covers all possible values for *B*. The maximal possible value of *B* is obviously the intersection between the two lines.

The net reaction rate of a reaction $\text{v}=v_{\text{f}}-v_{\text{r}}$ has its sign determined by ΔG, but establishing a direct relation beyond that is in general not possible. It has however been shown that the ratio between the two components $\log\left(v_{\text{f}}/v_{\text{r}}\right)$ is proportional to |ΔG|[23]. So even if an enzymatic reaction can keep the same flux for very different ΔG, an enzyme operating closer to equilibrium will be divided between catalyzing both the forward and reverse reaction, while the one operating far from it mostly catalyze the forward reaction. Thus, the stronger the driving force of the reaction is, the less amount of enzyme will be needed to hold the same flux. The cell would therefore make the most efficient use of it enzymes by keeping all its reactions as far from equilibrium as possible. One possible formulation of this principle has been proposed [24] as the Max-min Driving Force. A state, following this “design principle”, would be such that the less negative ΔG of the whole network—minimal driving force—is as far away from equilibrium as possible. In other words, enzyme efficiency can be achieved by ensuring that not a single reaction gets too close to equilibrium. This formulation offers the advantage that it can be written as a linear problem with help from Equation (4). This enables to obtain a solution as easily as in any FBA problem. Instead of writing it as an optimization problem, as was shown in detail in [22], we will just illustrate it graphically for a simple case. Let’s assume a simple metabolic pathway with only two reactions X1 → X2 → X3. Now, defining *B* as the ΔG of smallest magnitude—the less negative of all Gibbs free energies—Equation (4) will look like:

$$\begin{array}{c} g_{1}^{0}+R\;T\;\left(y_{2}-y_{1}\right)\;\leq \;B \end{array}$$

(5) $$\begin{array}{c} g_{2}^{0}+R\;T\;\left(y_{3}-y_{2}\right)\;\leq \;B \end{array}$$

*B* is, by definition, the minimal driving force. If we try to maximize its magnitude, it is easy to see that $y_{1}$ should be made as high as possible—within acceptable bounds—,since that would increase the overall ΔG of the pathway. The same reasoning shows $y_{3}$ should be set as low as possible. The second metabolite of the pathway $y_{2}$ has to find a compromise, since it splits the overall ΔG between the two reactions. Setting $y_{2}$ too high will hamper the first reaction, while setting it too low will hamper the second. Figure 3 shows how the ΔG of each reaction changes with $y_{2}$, one increasing and the other decreasing. Clearly, *B* must be below both curves (grayed area) and its maximum value is found for the concentration of $y_{2}$ such that $\Delta G_{1}=\Delta G_{2}$, as long as this point falls within the boundaries admitted for $y_{2}$. The effect of partitioning the total driving force among all the steps in the pathway brings us to another design principle, proposed thirty years ago [25] and recently confirmed to play a role in the architecture of metabolic pathways [26], the principle of metabolic simplicity [27]. According to this principle, a metabolic conversion will be carried out using the shortest pathway. The rationale for that is that if two pathways perform the same metabolic conversion, they will have the same overall ΔG. The shorter pathway will have a higher driving force for each step, carrying a higher flux per unit of enzyme activity. The relation between ΔG and rate will be explored in more detail in the next section.

### 2.3. Catalytic Efficiency of Enzymes

After sequential application of FBA and TFA, the system is described by a steady state flux distribution together with admissible intervals for ΔGs and metabolite concentrations. The rate laws of the enzymes bring these three sets of data together. Actually, just plugging all these rate laws in Equation (1) would yield a complete description of the system. However, the complexity of measuring all the necessary parameters is what motivated this work in the first place. In this section we will show that there are different techniques that provide a great deal of information without explicit knowledge of the rate equation and its parameters.

Practically all enzyme rates can be factored [22] as a constant term multiplied by a series of factors like:

(6) $$\text{v}\;=\;k_{cat}^{+}\;E_{T}\;\eta^{reg}\left(x\right)\;\eta^{sat}\left(x\right)\;\cdots \;\left(1-\theta \right)$$

**Figure 4:**
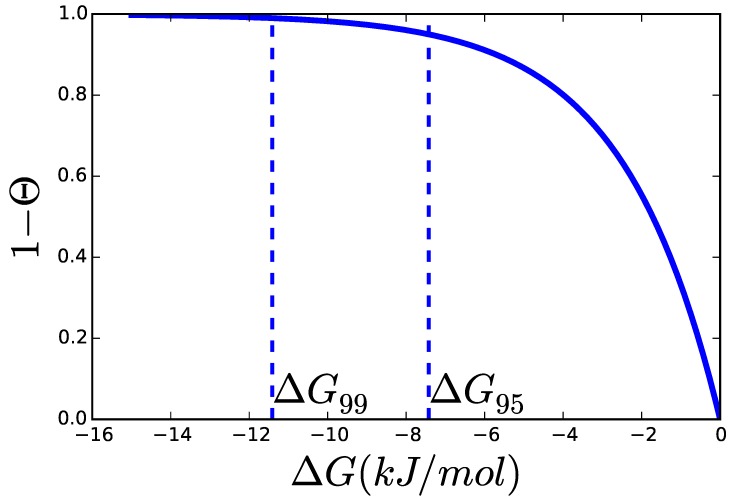
When ΔG is similar enough to RT (typically assumed to be 2.48 kJ/mol for biochemical reactions), the thermodynamic factor, 1−θ, decreases rapidly, since $\theta =exp(\frac{\Delta G}{R\;T})$. $\Delta G_{95\%}$ and $\Delta G_{99\%}$ are the affinities at which the enzyme can operate at 95% and 99% of its $V_{max}$ respectively.

The product of the catalytic constant $k_{cat}$ and the amount of enzyme $E_{T}$ is the maximal rate, $V_{max}$, so each of the other factors and the overall product of all of them are bounded between 0 and 1. Each of the $\eta^{k}$ functions accounts for a different effect of the metabolites on the reaction rate—e.g., allosteric inhibition, saturation, *etc.*—and depends also on a series of parameters, like Hill coefficients, saturation constants, *etc*. For some rate laws, it is difficult to factor allosteric and saturation terms separately, but it has been shown that the allosteric and other terms can always be factored out as a Hill-like term using a special type of Taylor series [28]. Finally, *θ* is the distance to equilibrium,

(7) $$\theta_{i}\;=\;\frac{\prod_{j}x_{j}^{s_{i,j}}}{K_{eq,i}}\;=\;\exp\left(\frac{\Delta G_{i}}{RT}\right)$$

and $s_{i,j}$ the stoichiometric coefficient with which $x_{j}$ participates in reaction *i*—positive for products and negative for substrates—and $K_{eq,i}$ is the apparent equilibrium constant. So we can write the thermodynamic factor (*θ*) as a function of the metabolites or just of the ΔG, which is already known from TFA. Even without knowledge of the concrete mechanism of a reaction, not to talk about the numerical value of its parameters, much can already be understood. For instance, a reaction operating at a certain distance to equilibrium has an effective maximal rate of $k_{cat}^{+}\;E_{T}\;\left(1-\theta \right)$. The effective $V_{max}$ increases monotonically but not linearly with the distance to equilibrium. Figure 4 shows this effect in terms of ΔG. In the following, we will use the notation $\Delta G_{x}$ for the ΔG at which the enzyme can operate up to x% of its $V_{max}$. The three factors, $k_{cat}^{+}$, $E_{T}$ and (1−θ), establish the minimal amount of enzyme that can sustain a certain flux [24]. Knowledge about the limits constraining these parameters and the values they are expected to adopt in living microorganisms is therefore of the greatest importance for the advancement of quantitative approaches to biochemistry.

#### 2.3.1. Theoretical Limits and Some Reference Values

Being the building blocks of metabolism, it is certain that enzymes must be the subject of some evolutionary pressure. It is however far from trivial to establish the magnitude of such pressure and in which direction it aims. Mutations will change the detailed behavior of the function presented above but which changes can be considered improvements from an evolutionary point of view? In many cases, the answer depends on the enzyme as well as the milieu in which the enzyme operates, in this context represented by the concentrations of substrate and product. For instance, if the enzyme is operating at saturation, its rate will be $k_{cat}^{+}\;E_{T}$, so it has been proposed that enzymes operating under such conditions will evolve towards a maximal $k_{cat}$ [29,30]. The opposite case, where substrate concentrations are far below the saturation constant, lead to linear rates, where $k_{cat}\;E_{T}/K_{M}$ is the proportionality constant, so this ratio would also be a reasonable target for evolutionary optimization [29,31,32]. The saturation constants themselves have been proposed to match metabolite concentrations [33], and additional goals have been defined [34,35]. According to theoretical calculations, diffusion imposes an upper bound for $k_{cat}$ of the order of $10^{10}\;s^{-1}$[36,37,38]. More recently, this view has been challenged [39] on the basis that many elementary steps involve processes that proceed on a slower timescale like conformational changes in the enzyme ∼$10^{6}$–$10^{8}\;s^{-1}$ or acid-base catalysis ∼$10^{6}\;s^{-1}$.

**Figure 5:**
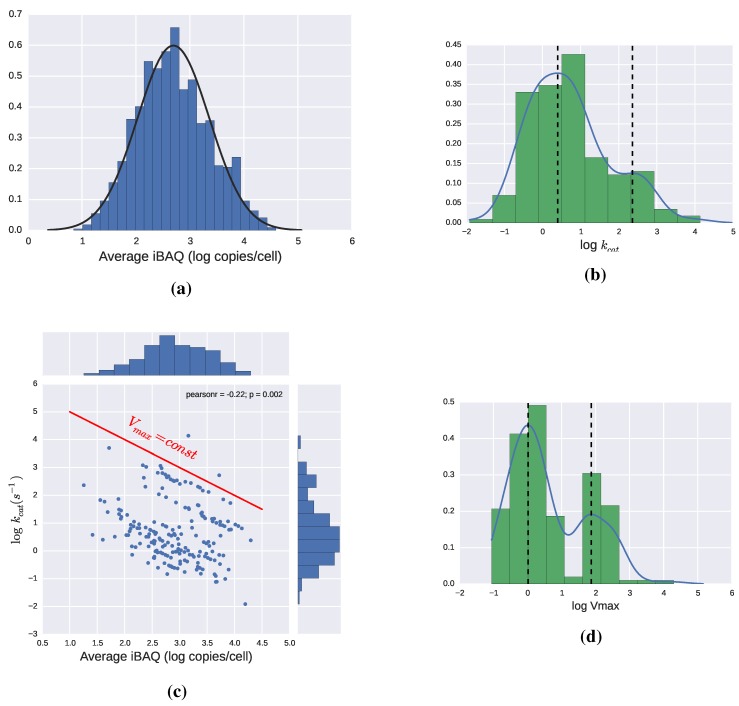
(**a**) Distribution of enzyme abundance in *E. coli* in copies/cell [40]; (**b**) Values of $k_{cat}$ for different enzymes. Bimodal distribution with modes 2.5 and 230 $s^{-1}$; (**c**) Values of $k_{cat}$ *vs.* copies per cell for different enzymes. In logarithmic coordinates, the product $k_{cat}\;E_{T}$, is a straight line, so a red line has been added as a reference. All enzymes falling in a line parallel to it have the same $V_{max}$; (**d**) Distribution of $V_{max}$. Bimodal distribution with modes approx 1 and 75 μM/s. Some histograms are complemented by Gaussian kernel density estimations performed using Python’s package Scipy.

While some enzymes, like Triosephosphate Isomerase, have lived up to the most optimistic theoretical calculations, the great majority of $k_{cat}$ values measured so far are well below the most conservative upper limits of $10^{6}\;s^{-1}$. By analyzing the data in the enzyme database BRENDA, Bar-Even *et al.* estimated the average enzyme to have a $k_{cat}$ of $10\;s^{-1}$[21]. Enzymes involved in central metabolic pathways were found to have an average value an order of magnitude higher, which still falls short of the theoretical predictions. The reasons for this discrepancy are still not known but two sorts of explanations have been proposed: (1) most enzymes have reached the point, where increasing their $k_{cat}$ conflicts with another goal, and this goal takes priority most of the time; (2) The evolutionary process is very slow due to different reasons like the low probability of some of the mutations required, diminishing returns from successive improvements or clonal interference between intermediary steps towards the improved phenotype.

The disagreement between theoretical limits and actual observations makes it especially relevant to develop a feeling for which values of $k_{cat}$ and enzyme levels are reasonable in microorganisms, since having a range for this values, and thus $V_{max}$, would be a first step towards kinetics. In this respect, examining the data from [40] can be particularly helpful, since they provide values of both enzyme concentrations and catalytic constants in *E. coli*. The expression of all the proteins in the cell follows a log-normal distribution, which is also appropriate to describe the subset of enzymes for which a catalytic constant value is provided, as can be seen in Figure 5a. The same data-set includes estimations for the catalytic constants of 190 enzymes, which, as can be seen in Figure 5b, have a certain tendency to follow bimodal distribution. The lack of correlation between $k_{cat}$ and enzyme levels has been reported in [21,40], and can clearly be seen in Figure 5c. By calculating $V_{max}$ for the enzymes in this dataset, we obtain again a bimodal distribution, shown in Figure 5d. This multimodality is consistent with the observations that point at enzymes from the central metabolism having higher catalytic efficiencies, than enzymes operating in the periphery of metabolism [21,40]. A more complete dataset may show additional modes but as a guideline for modeling we could conclude that there are a few groups of enzymes that roughly follow a log-normal distribution with the most frequent $V_{max}$ being around 1 and 75 μM/s. These very rough estimations should always be taken with a pinch of salt but can be a useful guide for the modeling process and smoothen the transition from flux distributions to more complex models.

### 2.4. Adding Regulation to Obtain a Dynamic Model

The approaches discussed so far have been extremely successful for two main reasons: First, they are based on linear equations. The simplicity and structural regularity of the linear formalism enables the use of very general techniques and its application to really big systems. Second, the amount of information necessary to set up a model is not too high and can be obtained in the omics scale. In spite of their undeniable usefulness, a very important feature is missing in both cases: regulation. Only introducing kinetics in the model can shed light into regulation and dynamics but identifying all the functions and parameters in Equation (6) is extremely complicated. Moreover, the non-linearity of kinetic reaction laws also precludes most analytical methods, forcing the researcher to use numerical approaches in which it is not clear what features of the model come from its regulation pattern and which features come from the specific kinetics used to model it. There is a need to look for methods that help identify key regulatory interactions without being tied to a specific choice of function shapes and parameter values [41]. It is therefore fortunate that many important properties can already be established using approximate kinetics, some based on non-linear approximations around the steady state—e.g., sensitivity analysis and dynamic simulations near the steady state—and some even based on linearizations—e.g., stability analysis. Such techniques enable the introduction of regulatory signals in the network even when the detailed mechanisms of the rate equations are not known. A link between sensitivities and thermodynamics has already been provided [24], and can be used as a stepping stone towards dynamic systems and using mathematically controlled comparison (MCC) to obtain general conclusions from partial information.

Reaction kinetics are non-linear, and linearizing them would result in unrealistic representations of the problem. The next best option is to obtain a linear representation in logarithmic coordinates.

(8) $$\ln\;{\text{v}}_{i}\;=\;\ln\;\alpha_{i}+g_{i,1}\;\ln\;x_{1}+g_{i,2}\;\ln\;x_{2}+\cdots +g_{i,n}\;\ln\;x_{n}$$

where each coefficient $g_{i,j}$ is the slope of the rate *vs.* the corresponding variable in a log-log plot and $\ln\alpha_{i}$ would be the intercept. In other words:

(9) $$g_{i,j}\;=\;\frac{\partial \ln\;{\text{v}}_{i}}{\partial \ln\;x_{j}}\;=\;\frac{\partial {\text{v}}_{i}}{\partial x_{j}}\frac{x_{j}}{{\text{v}}_{i}}$$

(10) $$\ln\;\alpha_{i}\;={\;\ln\;|\text{v}|}_{0}-\sum_{j}g_{i,j}\;\ln\;{\left|x_{j}\right|}_{0}$$

where the ${\left|x\right|}_{0}$ means the numerical value of *x* at the reference steady state. We can also undo the logarithmic transformation to obtain the following rate law:

(11) $${\text{v}}_{i}\;=\;\alpha_{i}\;x_{1}^{g_{i,1}}\;x_{2}^{g_{i,1}}\;\cdots \;x_{n}^{g_{n,1}}$$

The kind of rate in Equation (11) is known as power-law and it is the basis for developing fully dynamic models within Biochemical Systems Theory (BST) [7,8,9], see [42] for a thorough review. In such context, and by similarity with the mass action rate law, we will use the term kinetic orders for the *g* parameters and rate constants for the *α*s. Kinetic orders in a power law model, unlike in mass action kinetics, can have any real value. Kinetic orders are very often referred to as elasticities in the context of Metabolic Control Analysis (MCA) [43,44], where the power-law is used implicitly to perform sensitivity analysis. Any kind of sensitivity analysis done in one of these two sister disciplines can be easily translated to the other, but since BST is more oriented towards formulating and analyzing dynamic models, it provides additional tools that will be needed in this work. For this reason, we will use BST terminology consistently during the whole discussion and provide translations to the corresponding MCA concepts, when there is one.

**Figure 6:**
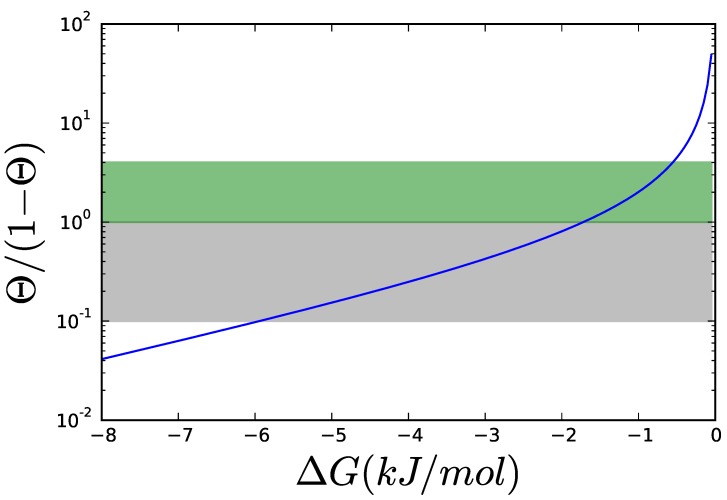
Thermodynamic contribution, $\frac{\theta }{1-\theta }$, to the kinetic order for different values of ΔG. The thermodynamic contribution is plotted in logarithmic coordinates. To simplify reading the curve, the area, where the thermodynamic contribution is between 0.1 and 1 is grayed. In this area, the thermodynamic contribution is comparable to the kinetic contribution, $g_{i,j}^{k}$ in Equation (12) for hyperbolic kinetics like MM, which varies between 0 and 1. Allosteric enzymes have kinetic contributions of magnitudes between 0 and 4, so an extra area for thermodynamic contributions between 1 and 4 has been marked.

Using separable reaction rates like Equation (6) as the starting point, and due to the properties of logarithms, each kinetic order becomes the sum of several contributions, one for each factor of the rate equation, including the thermodynamic factor [22]:

(12) $$g_{i,j}\;=\;g_{i,j}^{k}-s_{i,j}\frac{\theta }{1-\theta }$$

where the stoichiometric coefficient $s_{i,j}$ will be negative for substrates and positive for products. All the contributions to the kinetic orders due to non-thermodynamic effects—e.g., saturation and regulation terms—have been grouped in $g_{i,j}^{k}$. This kinetic term is bounded between 0 and 1 for Michaelis-Menten type kinetics and between 0 and the Hill coefficient for allosteric rate laws. The thermodynamic contribution is not bounded and will tend to infinity as the reaction approaches equilibrium (see Figure 6).

In order to do numeric analysis and simulations with such a system, the space of possible kinetic orders could be sampled using Monte Carlo methods but much can already be done without attributing numerical values to the parameters. This is a consequence of a well known property of systems like this (S-systems), namely that in spite of being fully non-linear, its steady states can easily be expressed as a system of linear equations in terms of the logarithms of its variables ($y_{i}=\ln x_{i}$) [8].

The availability of analytic solutions for the steady state brings the possibility of performing many different analysis symbolically without the need for numeric methods, like sensitivity analysis. Two kinds of sensitivities are normally defined: Logarithmic gains, $L_{x_{i},x_{j}}=\frac{\partial x_{i}}{\partial x_{j}}\frac{x_{j}}{x_{i}}$, $L_{{\text{v}}_{i},x_{j}}=\frac{\partial {\text{v}}_{i}}{\partial x_{j}}\frac{x_{j}}{{\text{v}}_{i}}$, measure the response of the system to changes in independent variables. Sensitivities to parameters $S_{x_{i},\alpha_{j}}=\frac{\partial x_{i}}{\partial x_{j}}\frac{x_{j}}{\alpha_{j}}$, $S_{{\text{v}}_{i},\alpha_{j}}=\frac{\partial {\text{v}}_{i}}{\partial x_{j}}\frac{x_{j}}{\alpha_{j}}$, measure the response of the system to changes in rate constants (or sometimes kinetic orders). The reasons for this distinction is that independent variables, regardless of their biological nature, normally represent the inputs/outputs/signals to which the system has to respond, while parameters normally reflect the composition of the system itself. Thus, logarithmic gains tend to indicate the responsiveness of the system, while parametric sensitivities indicate its robustness. The sensitivities to $\alpha_{i}$ are normally an indication of the response of the system to changes in the corresponding enzyme and are called control coefficients in MCA. The MCA counterpart for logarithmic gains can be a control or response coefficient, depending on the variable and how the system is defined.

Another consequence of the structural regularity of power-law models is that linearization is straightforward and the Routh-Hurwitz stability criterion [45] can be formulated in terms of the kinetic orders (see supplementary information). Especially important for the subsequent discussion will be the stability margin for the inhibition. For instance, it has been shown [3] that a strong feedback inhibition will make an unbranched metabolic pathway more robust but there is an upper limit, where the loop destabilizes the system. This limit can be established as a function of the parameters of the system.

(13) $$g_{1,3}<f\left(\alpha ,G\right)$$

### 2.5. Mathematically Controlled Comparison (MCC)

If we want to understand the kind of strategy that can be evolutionarily successful, we will have to compare alternatives. Comparing objects requires defining some criteria, according to which the objects can be ranked. Comparing classes or sets, however, is a much more complex problem, since two objects from the same class can rank different on the same criterion. Comparing the most honest and virtuous feudal lord with the a bunch of corrupt politicians may give the impression that the feudal system is preferable to a democracy [46]. So how can classes be ranked by judging the performance of their members? The solutions to this problem are many, since it is open-ended, but they all require a systematic approach. We are now interested in comparing different pathway designs so the first step must be a precise definition of the classes to be compared. Using power-law models makes it possible to define the classes very precisely—e.g., inhibition of the first reaction by the end product—without having to specify mechanistic details—is the inhibition allosteric or competitive?—thanks to the existence of analytical solutions like Equation (A7) for many magnitudes of interest. Finally, a protocol has to be established to generalize the results from comparing objects to compare classes. The method of MCC tackles this problem by defining a reference system to which different alternatives can be compared. The alternatives must satisfy two criteria of equivalence for the comparison to be meaningful:

**Internal equivalence** The reference and alternative system belong to different classes due to differences in one or more reactions/processes—e.g., the first reaction of the reference system is inhibited by the end product of the pathway, while the alternative system does not have this feedback loop. Since those distinctive reactions have been modified from one system to another, their parameters may differ. We will keep the notation, where, if *p* is a parameter or a property of the reference system, then $\hat{p}$ is the same parameter or property in the alternative system. For all the parameters not involved in distinctive reactions, internal equivalence is satisfied by making the parameters of every other reaction equal in both systems $p=\hat{p}$.

**External equivalence** Two systems satisfying the internal equivalence condition will have identical values for most parameters except for the handful involved in processes distinctive of their class. These degrees of freedom are further reduced by ensuring that both systems are perceived by their environments as being as similar as possible. That eliminates differences that are not inherent of the class but characteristic of some particular cases. Let’s imagine a pathway that has an optimal flux to provide a certain precursor for biomass. Introducing a feedback inhibition will decrease the flux through the whole pathway, making it suboptimal. Does it mean that the feedback is deletereous for the cell? Obviously not. The system with feedback is perfectly capable to supply the precursor in the same amounts, but that will require an increase in the activity of the inhibited enzyme. Only after setting the fluxes equal in both systems, can the comparison between the two alternative architectures be considered fair. An additional boon of this approach is that the degrees of freedom for the choices of parameter values is reduced, so the free parameters of the alternative system become a function of those of the reference system, and comparisons are made practically on a one to one basis. Typical requirements for external equivalence would be that both systems carry the same fluxes, have the same concentrations of initial and end product in the steady state, or that they have as many identical logarithmic gains as possible.

MCC provides clear cut analytical solutions, when a systemic property is always better in one of the alternatives being compared or when both alternatives are always identical. In cases, where a quantitative prediction is wanted or when there is a trend for one system to outperform the other, a statistical approach to MCC can be followed [47].

## 3. Results

The use of constraint based approaches as a stepping stone towards dynamic models has so far been limited to stoichiometric constraints [48,49,50]. Now we will show a systematic method to include TFA as an intermediary step. In subsequent sections, three case studies will be presented as proof of concept of the advantages of the FBA/TFA/BST workflow. In case study one, FBA and TFA will be applied successively to a well studied and simple system: ammonia assimilation in bacteria. The results on this system, for which a large body of knowledge has been accumulated, should serve as a guide for not so well known and more complex systems. Case study 2 will deal with another well studied system: the unbranched pathway with feedback inhibition. This example will show how the incorporation of thermodynamic considerations to a dynamic model can lead to the discovery of a new design principle. Finally, case study 3 will integrate the new and the old results on unbranched pathways to propose a hypothesis on how design principles that concern different levels of study: stoichiometric, thermodynamic and kinetic can be combined to propose two alternative strategies for the evolution or synthetic design of metabolic pathways.

As has been shown above, the proximity to equilibrium has an influence on the values of the kinetic orders. For reactions far away from equilibrium, ΔG>>RT, this influence can be ignored and the kinetic orders of the system can safely be determined independently of the thermodynamics. But when the steady state, chosen as a reference for the definition of the model, has reactions close to equilibrium, thermodynamic considerations must be brought into the modeling process. The distances of the reactions to equilibrium, $\theta_{i}$, in the reference state are introduced as parameters in the definition of the system. The new parameters must be introduced at three levels. First, in the definition of the kinetic orders as shown in Equation (12). Second, since $\theta_{i}$ is the proximity to equilibrium in the reference steady state, the steady state solutions for the metabolites must satisfy the definition of *θ* provided in Equation (7):

(14) $$\prod_{j}{\left|x_{j}\right|}_{0}^{s_{i,j}}\;=\;\theta_{i}\;K_{eq,i}$$

Finally, if we intend to use *θ* in mathematically controlled comparison, we will have to take it into account, when the equivalence conditions are established (see case study 2 for an example).

**Thermodynamic shortening** As has been shown above, as a reaction approaches equilibrium, its rate becomes hypersensitive to perturbations of substrates and products, their kinetic orders tending to infinite magnitude in the sense that counters the perturbation, positive for substrate and negative for product. Also, by definition, the mass action quotient will tend to the value of the equilibrium constant. This enables to reformulate the model in a reduced form that will, nevertheless, be consistent with the behavior of the full metabolic network, as has been established using perturbation theory [51].

The first step is to establish which reactions are close to equilibrium. Then, we can partition the fluxes of the system into those close to equilibrium, $v_{\mathrm{eq}}$, and those far from equilibrium, $v_{\mathrm{irr}}$, and convert Equation (1) into:

(15) $$\dot{x_{d}}\;=\;S_{\mathrm{eq}}\;v_{\mathrm{eq}}\left(x\right)+S_{\mathrm{irr}}\;v_{\mathrm{irr}}\left(x\right)$$

It is important to note that the vector of metabolites on the right hand side of the equation contains the full vector x, since fixed metabolites will be defined as independent variables, and matrix S has to be defined accordingly.

Now, the mass action quotient will constrain the metabolite concentrations. Writing Equation (14) in matrix form:

(16) $$\log\left(k_{\mathrm{eq}}\right)\;=\;S_{\mathrm{eq}}^{T}\;\log\left(x\right)$$

where $k_{\mathrm{eq}}$ is a vector containing the equilibrium constants of the reactions close to equilibrium.

Now, we can define pools of metabolites in equilibrium.

(17) p=Cx

These pools will be enough to characterize all the variables of the system, since the concentration of each metabolite in a pool can be obtained from the mass in the pools and Equation (16). For this to be the case, pools must be defined, such that the fluxes $v_{\mathrm{eq}}$ from Equation (15) vanish from the new set of equations, since they become internal fluxes within the pools. This can be achieved by choosing C according to the strategy defined by Gerdtzen *et al.* [51]. The differential equations can be written as follows:

(18) $$\dot{p}\;=\;C\;\dot{x}\;=\;C\;S_{\mathrm{eq}}\;v_{\mathrm{eq}}\left(x\right)+C\;S_{\mathrm{irr}}\;v_{\mathrm{irr}}\left(x\right)$$

By choosing C from the left-null space of $S_{\mathrm{eq}}$, we will ensure that near equilibrium fluxes will vanish:

(19) $$\dot{p}=C\;S_{\mathrm{irr}}\;v_{\mathrm{irr}}\left(x\right)$$

Since Equation (16) creates dependencies among the metabolites, we can partition the metabolites in two sets: free metabolites $x_{f}$ and bound metabolites $x_{b}$, whose concentrations will be determined by the free ones and the constraints.

(20) $$\log\left(k_{\mathrm{eq}}\right)=-S_{\mathrm{eq},f}^{T}\;\log\left(x_{f}\right)+S_{\mathrm{eq},b}^{T}\;\log\left(x_{b}\right)$$

There are a number of possible partitions, all of them valid as long as $S_{\mathrm{eq},b}^{T}$ is invertible. Then we can eliminate the bound metabolites, since they are a function of the free ones.

(21) $$\log\left(x_{b}\right)\;=\;-{\left(S_{\mathrm{eq},b}^{T}\right)}^{-1}\;S_{\mathrm{eq},f}^{T}\;\log\left(x_{f}\right)+{\left(S_{\mathrm{eq},b}^{T}\right)}^{-1}\;\log\left(k_{\mathrm{eq}}\right)$$

Equation (21) is a power-law, so it can easily be used to eliminate $x_{b}$ from any power-law model and yet preserve the structure of the system.

Rewriting the equations of the system,

(22) $$\begin{array}{cc} \dot{p}\;= & \;C\;S_{\mathrm{irr}}\;v_{\mathrm{irr}}\left(x\right) \end{array}$$

(23) $$\begin{array}{cc} x_{b}\;= & \;\mathrm{diag}\left(\gamma \right)\;x_{f}^{F} \end{array}$$

where $\gamma =k_{\mathrm{eq}}^{{\left(S_{\mathrm{eq},b}^{T}\right)}^{-1}}$ and $F={\left(S_{\mathrm{eq},b}^{T}\right)}^{-1}\;S_{\mathrm{eq},f}^{T}$.

The change of variables is not yet complete, since Equation (23) still contains the old and the new variables. In simple systems, completing the change by eliminating x is a trivial substitution, while more complex cases will result into a system of differential-algebraic equations due to the additional constraints. In any case, steady state analysis can always be performed directly from Equation (23), since in the steady state, $\dot{p}$ and the set of equations become a set of algebraic equations in terms of only $x_{f}$.

### 3.1. Case Study 1: Ammonia Assimilation

**Figure 7:**
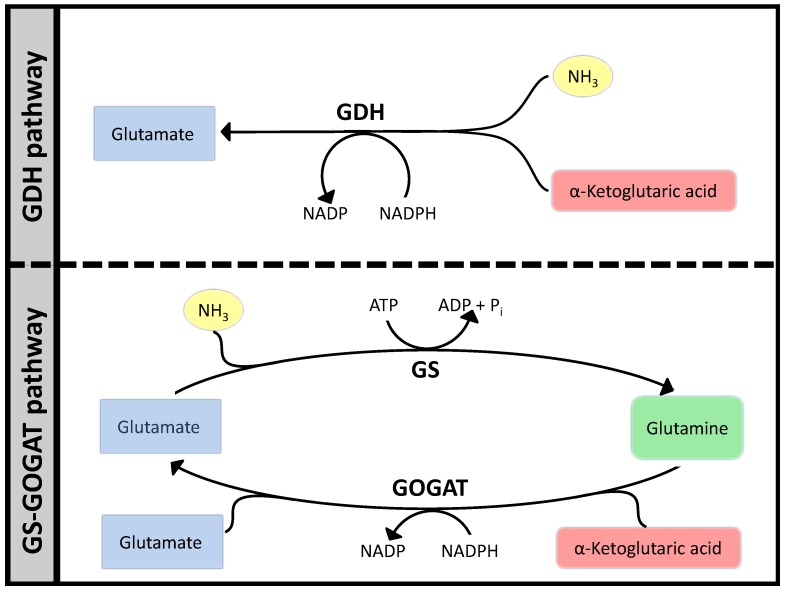
Two pathways for ammonia assimilation.

Ammonia assimilation follows a very similar pattern in many bacteria, based on two parallel systems: Glutamate Dehydrogenase (GDH) that catalyzes the reductive amination of *α*-keto-glutaric acid to glutamate and the tandem GS/GOGAT that starts with the amination of glutamate to glutamine by Glutamine Synthase (GS) and then proceeds to transfer the NH_3_ to *α*-ketoglutaric acid by Glutamate Synthase (GOGAT). The sum of these two reactions are equivalent to the action of GDH plus the hydrolysis of one ATP. Viewing this small network (see Figure 7) from a stoichiometric perspective, would show two Elementary Flux Modes, one for GDH and another for GS/GOGAT. Optimizing a bigger network using FBA will often result in GDH as the optimal path to carry the flux, since that would free ATP for other pathways. It is however well known that GS/GOGAT is the preferred pathway for nitrogen assimilation with the GS/GOGAT system being very strictly controlled at many levels. In fact, the whole set of regulatory processes around this simple set of reactions is extremely complex, see [52] for a thorough review. However, focusing our attention in the biochemistry can help us understand much about the constraints that have conditioned the evolution of the system. Complementing FBA with additional analysis presented above and interpreting the result from the point of view of “design principles” will already clarify things significantly.

The longer pathway needs two enzymes, that is twice as much protein assuming all enzymes have the same $k_{cat}$ and are working at saturation. Moreover, the GS/GOGAT system needs one ATP more, which is the only feature captured at the Flux Balance Analysis (FBA) level and often results in FBA solutions favoring glutamate dehydrogenase (GDH).

**Figure 8:**
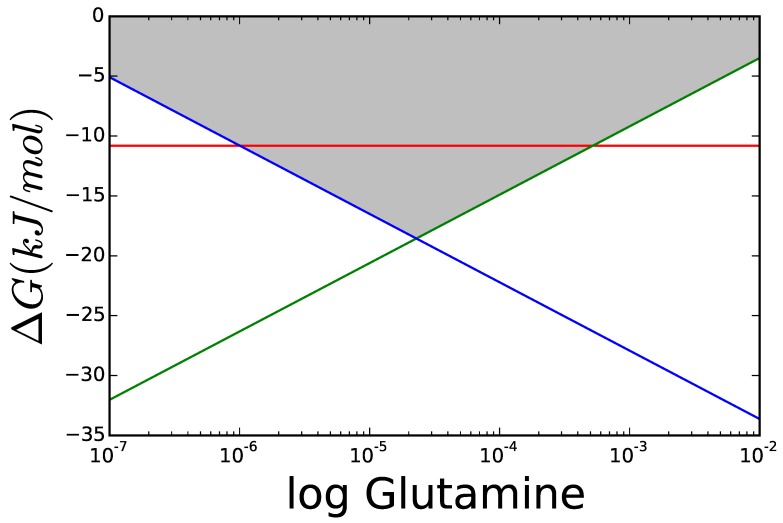
The lines represent the ΔG of each reaction at different concentrations of glutamine for a fixed NH_3_ = 1 mM. Concentrations of other metabolites taken from the literature (see supplementary information), except *α*-ketoglutaric acid that was set as high as the default boundary permitted. Red GDH, green GS and blue GOGAT.

Figure 8 shows ΔG for each of the three reactions at different concentrations of glutamine, using reasonable values for the metabolites involved taken from the literature [6,20] and thermodynamic data taken from the Equilibrator software [53] (see supplementary information). GDH does not depend on glutamine but the two reactions of the GS/GOGAT pathway behave as in the previous example, with glutamine determining how the overall ΔG of the pathway is split between its two reactions. Applying the concept of Max-min Driving Force discussed above, it is clearly seen that each of the two reactions in the long pathway can have higher driving forces than GDH! This difference will depend on the amount of NH_3_ available, as we’ll see below.

**Figure 9:**
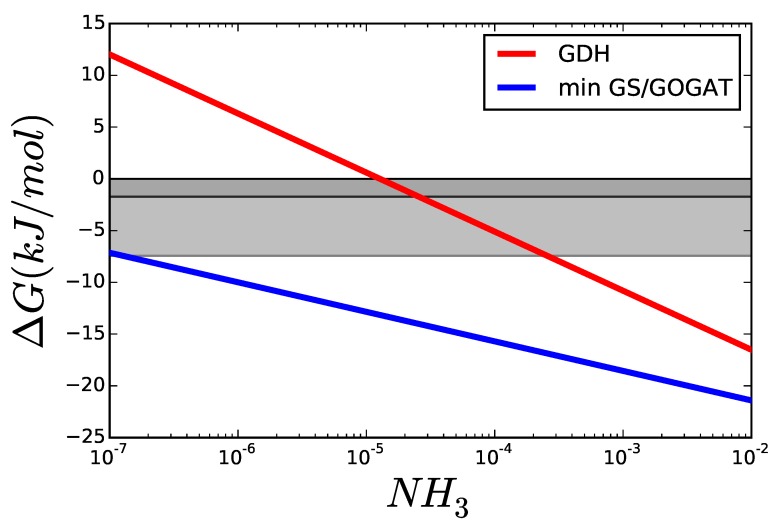
Comparison of ΔG of GDH and GS/GOGAT applying the Max-min Driving Force principle so that both GS and GOGAT have the same ΔG. The greyed areas mark values, where ΔG is similar enough to RT that the thermodynamic factor 1−θ decreases rapidly. The darker area marks $\Delta G_{50\%}$, and the lighter $\Delta G_{95\%}$.

The optimal glutamine concentration was calculated for varying the cytoplasmic concentration of NH_3_, while all other variables were kept equal to those in Figure 8. Figure 9 shows the resulting ΔG for each reaction. For low concentrations of nitrogen, GDH is thermodynamically unable to carry any flux and its driving force remains below that of GS and GOGAT. The area marked in grey shows scenarios, where the enzyme can operate but, due to its proximity to equilibrium, will need more enzyme to carry the flux, than each of the other reactions. Even if all enzymes had the same $V_{max}$, a GDH operating under $\Delta G_{50\%}$—dark grey area—would have to be present in a concentration higher than the sum of the other two enzymes to carry the same flux.

By sequential application of FBA and TFA it is clearly seen why the two parallel pathways are needed and which is more appropriate under which conditions. In this case, we are dealing with a well known pathway, so it is known that the GS/GOGAT system has low saturation constants, while the GDH enzyme has higher saturation constants, so it can progressively take over the flux as nitrogen concentration in the cell is high enough. This also shows how GS/GOGAT is a clear adaptation to environments poor in ammionia and the use of this pathway can lead to higher costs, than needed for nitrogen assimilation in a nitrogen rich environment. This has been shown overexpressing GDH from *E. coli* in a glutamate GOGAT deficient mutant of *Methylopilus methylotrophus*. The resulting strain was able to grow 4%–7% more in media containing high levels of ammonia [54].

### 3.2. Case Study 2: Thermodynamic Shortening of an Unbranched Pathway

We will now show how to transition from constraint based to dynamic models using the simplest possible metabolic pathway as an example, although the same principles can be used for systems of different nature and complexity [3]. A compendium of relevant previous results on this model [3,55], reworked under an unified notation, can be found in the supplementary information. Let’s assume an unbranched pathway from metabolite $X_{0}$ to $X_{3}$ with end-product inhibition. 
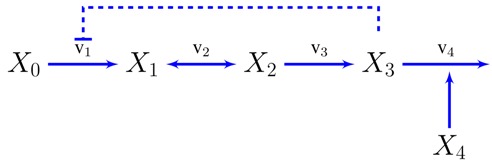


The concentration of the first metabolite is assumed to be held constant and $x_{4}$ is a variable representing the demand for the metabolite $X_{3}$, so $x_{0}$ and $x_{4}$ will be independent variables in the sense discussed in the introduction.

Since the stoichiometric information is the starting point for FBA, we can already set up our equations in terms of the fluxes:

$$\begin{array}{cc} {\dot{x}}_{1}= & \;{\text{v}}_{1}-{\text{v}}_{2}\; \end{array}$$

(24) $$\begin{array}{cc} {\dot{x}}_{2}= & \;{\text{v}}_{2}-{\text{v}}_{3}\; \end{array}$$

$$\begin{array}{cc} {\dot{x}}_{3}= & \;{\text{v}}_{3}-{\text{v}}_{4} \end{array}$$

The reaction rates in power-law form can already be written, at least symbolically, from inspection of the pathway map. Every variable influencing a reaction, through mass-action or otherwise, will get a kinetic order and it will be positive or negative depending on whether the variable activates or inhibits the reaction. For instance, ${\text{v}}_{2}$ and ${\text{v}}_{3}$ will only depend on their substrates and products: ${\text{v}}_{i}=\alpha_{i}\;x_{i-1}^{g_{i,i-1}}\;x_{i}^{g_{i,i}}$. ${\text{v}}_{1}$ and ${\text{v}}_{4}$ will be assumed to be unaffected by their product concentrations, the first will have a (negative) kinetic order accounting for the end product inhibition ${\text{v}}_{1}=\alpha_{1}\;x_{0}^{g1,0}\;x_{3}^{g1,3}$ and the second will depend on the demand ${\text{v}}_{4}=\alpha_{4}\;x_{3}^{g4,3}\;x_{4}$. Now we can write the dynamic model as a system of differential equations.

$$\begin{array}{cc} {\dot{x}}_{1}= & \;\alpha_{1}\;x_{0}^{g_{1,0}}\;x_{3}^{g_{1,3}}-\alpha_{2}\;x_{1}^{g_{2,1}}\;x_{2}^{g_{2,2}}\; \end{array}$$

(25) $$\begin{array}{cc} {\dot{x}}_{2}= & \;\alpha_{2}\;x_{1}^{g_{2,1}}\;x_{2}^{g_{2,2}}-\alpha_{3}\;x_{2}^{g_{3,2}} \end{array}$$

$$\begin{array}{cc} {\dot{x}}_{3}= & \;\alpha_{3}\;x_{2}^{g_{3,2}}-\alpha_{4}\;x_{3}^{g_{3,4}}\;x_{4} \end{array}$$

The particular steady state around which the system will be modeled can come from the previous constraint based analysis. FBA can provide the values of the fluxes, $|{\text{v}}_{i}{|}_{0}$ (see Figure 1). The concentrations of metabolites can be chosen within the range of feasible values provided by TFA, taken from a particular solution of the thermodynamic feasibility problem—e.g., Max-min Driving Force—, experimentally measured or a combination of all of the above. Once the steady state is identified, what remains to obtain a dynamic model are the parameters: kinetic orders and rate constants. Of these two sets of parameters, the latter can be determined from the former, as shown in Equation (10). About the kinetic orders, much is already known. All the signs are determined: $g_{1,0}>0$, $g_{1,3}<0$, $g_{i,i}\leq 0$ and $\;g_{i-1,i}\geq 0\;\;\forall \;i$. It is also known, that for reactions close to equilibrium, the kinetic orders will be dominated by their thermodynamic term $\theta_{i}\to 1\;\Rightarrow \;g_{i-1,i}≃-g_{i,i}\gg 1$ whereas far from equilibrium $\theta_{i}\to 0\;\Rightarrow \;g_{i-1,i}=g_{i-1,i}^{k}\;,\;g_{i,i}=g_{i,i}^{k}$. The strictly kinetic contributions have clearly defined bounds, their absolute value being below one for hyperbolic kinetics and below the Hill coefficient for allosteric. Finally, for the particular case of fully irreversible kinetics, the system can be fully simplified by setting $g_{i,i}=0$.

**Reactions near equilibrium** Thermodynamic analysis has established that reactions operating close to equilibrium originate a higher cost in terms of enzyme. Now, the question remains: can they also provide any advantage?

The effect of thermodynamics can be investigated by analyzing a pathway, where all the reactions are fully irreversible θ=0, except one, in our example $v_{2}$, which will be at an arbitrary distance to equilibrium *θ*. That way, the system properties can be analyzed as functions of *θ*.

Under these conditions, the steady state concentrations of the substrate and product of reaction 2 will be bound by Equation (14), in logarithmic form:

(26) $$|y_{2}{|}_{0}-{\left|y_{1}\right|}_{0}=\ln\left(\theta \;K_{eq}\right)$$

but also, from ${\dot{x}}_{2}=0$

(27) $$|y_{2}{|}_{0}\;=\;\frac{b_{2}}{g_{2,2}-g_{3,2}}-\frac{g_{2,1}}{g_{2,2}-g_{3,2}}\;{\left|y_{1}\right|}_{0}$$

just by equating coefficients in both expressions:

(28) $$\begin{array}{cc} -\frac{g_{2,1}}{g_{2,2}-g_{3,2}}\;= & \;1 \end{array}$$

(29) $$\begin{array}{cc} \frac{b_{2}}{g_{2,2}-g_{3,2}}\;= & \;\ln(K_{eq}\;\theta ) \end{array}$$

Equation (28) can be further simplified to:

(30) $$g_{3,2}=g_{2,1}+g_{2,2}$$

where the thermodynamic terms of substrate and product are equal and of opposite sign, so they cancel out:

(31) $$g_{3,2}=g_{2,1}^{k}+g_{2,2}^{k}$$

using this and $b_{2}=\ln\alpha_{3}-\ln\alpha_{2}$ (see supplementary information), Equation (29) becomes:

(32) $$\ln\alpha_{2}\;=\;\ln\alpha_{3}-\left(g_{2,1}^{k}+\frac{\theta }{1-\theta }\right)\;\ln\left(K_{eq}\;\theta \right)$$

Equations (31) and (32) enforce a special type of equivalence constraints on kinetic parameters needed for thermodynamic consistency. It is known that thermodynamics impose constraints on kinetic parameters and detailed formalisms have been proposed to include them in the formulation of dynamic models, when kinetics are known [56]. The expressions above show how thermodynamic constraints on kinetics can be even obtained without detailed knowledge of the kinetics involved. Including such thermodynamic requirements in kinetic models enables a further reduction of degrees of freedom in the estimation problem.

**Equivalence conditions** Due to internal equivalence, only the parameters involved in the second reaction can change with *θ*, which leaves four degrees of freedom: *θ*, $g_{1,2}^{k}$, $g_{1,2}^{k}$ and $\alpha_{2}$.

The external equivalence conditions that all the fluxes, as well as $x_{3}$, remain the same for all *θ* are automatically fulfilled, since neither the concentration of $x_{3}$ in the steady state nor the flux through the pathway depend on *θ*. So no reduction in the degrees of freedom comes from those conditions. The thermodynamic conditions in Equations (31) and (32), reduce the degrees of freedom by two.

(33) $${\hat{g}}_{2,2}^{k}=g_{3,2}-{\hat{g}}_{2,1}^{k}$$

(34) $${\hat{g}}_{2,1}^{k}\;=\;\frac{\ln\alpha_{3}-\ln{\hat{\alpha }}_{2}}{\ln(K_{eq}\;\theta )}-\frac{\theta }{1-\theta }$$

To eliminate the last degree of freedom we start writing the rate law for the reaction:

(35) $$|v_{2}{|}_{0}\;=\;\alpha_{2}\;{|x_{1}{|}_{0}}^{g_{2,1}}\;{|x_{2}{|}_{0}}^{g_{2,2}}$$

Introducing the thermodynamic formulation of the kinetic orders from Equation (12) and rearranging terms:

(36) $$|v_{2}{|}_{0}\;=\;\alpha_{2}\;{|x_{1}{|}_{0}}^{g_{2,1}^{k}}\;{|x_{2}{|}_{0}}^{g_{2,2}^{k}}\;{\left(\frac{|x_{2}{|}_{0}}{|x_{1}{|}_{0}}\right)}^{-\frac{\theta }{1-\theta }}$$

or

(37) $$|v_{2}{|}_{0}\;=\;\alpha_{2}\;{|x_{1}{|}_{0}}^{g_{2,1}^{k}}\;{|x_{2}{|}_{0}}^{g_{2,2}^{k}}\;{\left(\theta \;K_{eq}\right)}^{-\frac{\theta }{1-\theta }}$$

When θ<<1, the thermodynamic contribution will become irrelevant,

(38) $$\lim_{\theta \to 0}{\left|v_{2}\right|}_{0}\;=\;\alpha_{2}\;{|x_{1}{|}_{0}}^{g_{2,1}^{k}}\;{|x_{2}{|}_{0}}^{g_{2,2}^{k}}$$

and the system will revert to the far from equilibrium case analyzed in [3], where only the kinetic contribution to the kinetic orders is relevant. So the extreme case, where *θ* is negligible, can be taken as a reference and then equivalence conditions can be established.

(39) $$\hat{\alpha_{2}}\;=\;{\left|\alpha_{2}\right|}_{\theta =0}\;{\left(\theta \;K_{eq}\right)}^{-\frac{\theta }{1-\theta }}$$

where ${\left|\alpha_{2}\right|}_{\theta =0}$ would be the value of $\alpha_{2}$ in a fully irreversible model. This relation reflects the necessity of using more enzyme to keep the flux as the reaction approaches equilibrium.

**Systemic properties** Once the equivalence conditions have been set, the behavior of the pathway, when a reaction approaches equilibrium, can be checked analytically by taking limits of the systemic properties, when $\theta \to 1^{-}$. As a reaction approaches equilibrium, the kinetic orders for both substrate and product tend to plus and minus infinity respectively, attesting for an extremely fast response to any deviation. This hypersensitivity of the reaction comes together with a desensitization of the pathways to the reaction (Logarithmic gains/Control Coefficients tend to zero [24]),

(40) $$\lim_{\theta \to 1^{-}}S_{x_{j},\alpha_{2}}=0\;\forall j$$

so the influence of the enzyme in the overall steady state of the pathway vanishes.

The response of the pathway to supply and demand remains the same, since most logarithmic gains do not depend on *θ* and for those that do:

(41) $$\lim_{\theta \to 1^{-}}L_{x_{i},x_{j}}\;=\;\frac{g_{2,1}}{g_{3,2}-g_{2,2}}\;{\left|L_{x_{i},x_{j}}\right|}_{\theta =0}\;j\in \left\{0;4\right\}$$

where the factor has already shown to be thermodynamically constrained to be 1, so all logarithmic gains can be made equal between the two alternatives.

**Thermodynamic shortening** It has been shown that pathways with feedback inhibition tend to have a narrower margin of stability—right hand side of Equation (13)—as they grow longer [3], see also supplementary information. Short pathways can have really strong feedback loops, while longer ones must keep the strength of the inhibition signal weaker or risk becoming unstable. The stability of a long pathway is improved, when the kinetic parameters of its enzymes are distributed along a wide interval, such that each enzyme has very different kinetics. This phenomenon, called kinetic shortening, has been observed in long aminoacid synthesis pathways and could be considered a design principle in its own right. Now we will show how bringing some reactions close to equilibrium can have a similar effect that, by analogy, we will call thermodynamic shortening.

When a reaction of the system, in this case $v_{2}$, remains close to equilibrium, then its substrate and product will be very close to their equilibrium ratio $x_{2}≃K_{eq}\;x_{1}$. The relevant variable now is no longer $x_{1}$ or $x_{2}$, but the pool they form $p_{1}=x_{1}+x_{2}$[51]. The differential equation for the pool can easily be obtained from its definition: ${\dot{p}}_{1}={\dot{x}}_{1}+{\dot{x}}_{2}$. The new system will be:

(42) $$\begin{array}{cc} {\dot{p}}_{1}\;= & \;\alpha_{1}\;x_{0}^{g_{1,0}}\;x_{3}^{g_{1,3}}-\alpha_{3}\;x_{2}^{g_{3,2}}\;x_{3}^{g_{3,3}}\; \end{array}$$

(43) $$\begin{array}{cc} {\dot{x}}_{3}\;= & \;\alpha_{3}\;x_{2}^{g_{3,2}}\;x_{3}^{g_{3,3}}-\alpha_{4}\;x_{3}^{g_{3,4}}\;x_{4} \end{array}$$

Now the old variables must be eliminated. We combine the equilibrium relation with the definition of the pool, ${\dot{p}}_{1}=\left(1+K_{eq}\right){\dot{x}}_{1}$, so both $x_{1}$ and $x_{2}$ can be written as a function of the pool.

(44) $$\begin{array}{cc} {\dot{p}}_{1}\;= & \;\alpha_{1}\;x_{0}^{g_{1,0}}\;x_{3}^{g_{1,3}}-\alpha_{3}{\left(\frac{K_{eq}}{1+K_{eq}}\right)}^{g_{3,2}}\;p_{1}^{g_{3,2}}\;x_{3}^{g_{3,3}}\; \end{array}$$

(45) $$\begin{array}{cc} {\dot{x}}_{3}\;= & \;\alpha_{3}{\left(\frac{K_{eq}}{1+K_{eq}}\right)}^{g_{3,2}}\;p_{1}^{g_{3,2}}\;x_{3}^{g_{3,3}}-\alpha_{4}\;x_{3}^{g_{3,4}}\;x_{4} \end{array}$$

Defining ${\bar{\alpha }}_{3}=\alpha_{3}\;{\left(\frac{K_{eq}}{1+K_{eq}}\right)}^{g_{3,2}}$ results in

(46) $$\begin{array}{cc} {\dot{p}}_{1}\;= & \;\alpha_{1}\;x_{0}^{g_{1,0}}\;x_{3}^{g_{1,3}}-{\bar{\alpha }}_{3}\;p_{1}^{g_{3,2}}\;x_{3}^{g_{3,3}}\; \end{array}$$

(47) $$\begin{array}{cc} {\dot{x}}_{3}\;= & \;{\bar{\alpha }}_{3}\;p_{1}^{g_{3,2}}\;x_{3}^{g_{3,3}}-\alpha_{4}\;x_{3}^{g_{3,4}}\;x_{4} \end{array}$$

In this particular case, the margin of stability of the starting pathway was wide due to the small number of intermediates but becomes infinity after the reduction. If S(θ) is a systemic property of the original system—e.g., log-gain—and $\bar{S}$ is the corresponding property in the shortened system, then $\lim_{\theta \to 1^{-}}S\left(\theta \right)=\bar{S}$. This procedure can easily be shown to be general enough to be applied to a pool of several metabolites in a pathway of length *n*, *de facto* shortening it.

### 3.3. Case Study 3: Two Alternative Designs for an Unbranched Pathway

The unbranched pathway with end-product inhibition illustrates the concept of archetypes as defined by Shoval *et al.* [2]. On the one hand, following the principle of Max-min Driving Force, with enzymes working at saturation and low concentration of inhibitor, can provide highest flux for a certain investment in enzymes (economic design). On the other hand, an alternative pathway design can favor performance (responsive design). This can be achieved when the pathway has a first irreversible step and subsequent steps close to equilibrium. Furthermore, by way of kinetic and thermodynamic shortening, the margin of stability for this pathway can be increased. The importance of this margin does not rest on stability *per se*; increasing the margin means that the feedback loop can be several times stronger without causing instability, making the shortened pathway even more robust and responsive.

**Figure 10:**
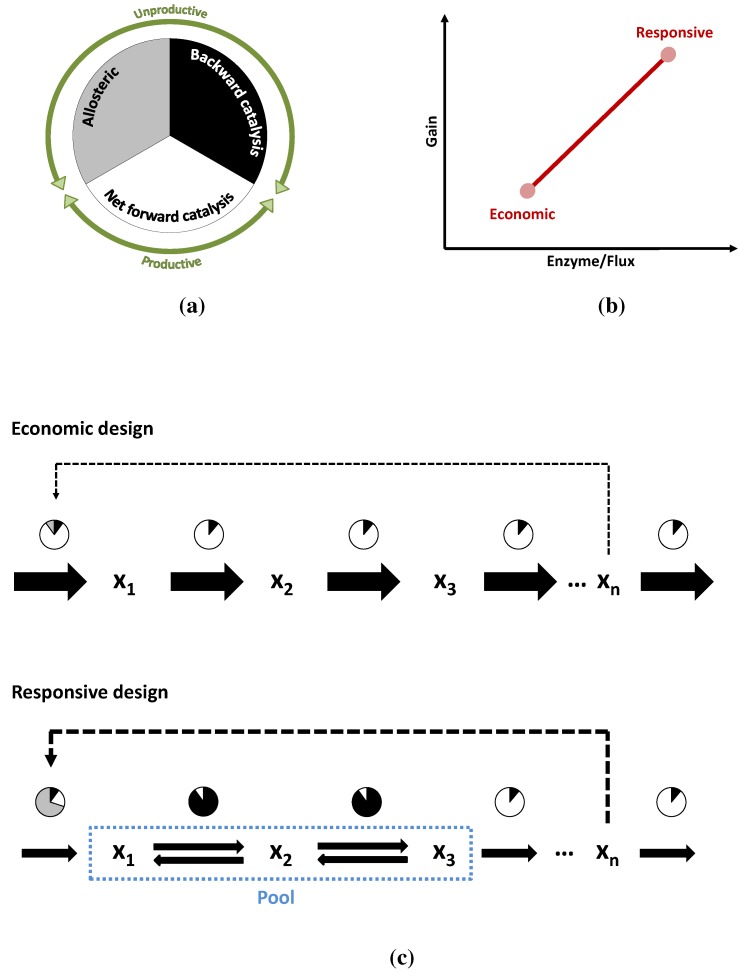
The circles in (**a**,**c**) schematically show the reduction of enzyme activity due to different causes (graphical depiction of the *η* terms in Equation (6)). In white, the fraction of the enzyme that is actually catalyzing the reaction forward. In grey the fraction that is inactive due to allosteric inhibition (or more exactly the fraction in which the total activity is reduced). In black, the fraction of activity that is lost due to the flux of the reverse reaction or due to insaturation of the enzyme; (**b**) Shows the two alternative pathways in performance space and the trade-off between them; (**c**) A more detailed depiction of the two modes of operation: The economic variant carries a high flux and all its enzymes are operating close to their $V_{max}$ due to weak inhibition and distance to equilibrium. In the responsive variant, the first enzyme is inhibited and two of the reactions are close to equilibrium. The efficiency of the enzymes is much lower but so is the flux they have to carry.

Comparing Figure 4 and Figure 6 makes it clear that keeping reactions close to equilibrium is no free lunch. The range of energies, where closeness to equilibrium makes a kinetic difference, may well be at or below $\Delta G_{10\%}$, so the performance of the responsive pathway must be paid for. The same happens with allosteric inhibitions, which also reduce the amount of “working” enzyme at any given time. As in the case of Darwin’s finches, these two alternatives need not be mutually exclusive. A pathway may operate well somewhere between both points depending on its metabolic niche, since they can be understood as extremes in a trade-off. But the point of operation is not necessarily fixed, pathways can alternate between both options, as shown in Figure 10a–c, moving from one mode of operation to another. Under conditions of low demand, the flux through the pathway is low enough that it can be kept with reasonable amount of enzymes, even if they operate at only a fraction of their $V_{max}$. Also under such conditions, it is important for the cell that the pathway is prepared to increase production as swiftly as possible, when demand increases. Such a stand-by state is the perfect scenario for the responsive design. As the flux through the pathways increases, substrate concentrations can naturally fall down following a staircase pattern, bringing the reactions further away from equilibrium towards a more economic mode of operation. Enzymes will then carry flux much more efficiently due to thermodynamics and also due to the lower concentration of inhibitor. Upon reaching the maximum demand, the pathway will have walked the Pareto line from responsive to economic.

## 4. Conclusions

Biology seems to be the science of exceptions, a bazaar offering a thousand different solutions for every problem. But the continuous recurrence of certain patterns is as characteristic of biology as diversity itself. The same counter current mechanism that concentrates urine in the kidney serves as a heat saving mechanism in the feet of the penguins. These patterns can also find their way across levels of organization: feedback inhibition is ubiquitous in the nervous system but it is also a fixture in metabolic pathways as we have discussed above. In this work, we have tried to show that the recurrence of some patterns are often the product of evolution. These patterns can, not only be recognized, but also predicted from first principles. Few problems have a unique solution and some problems have several that are equally good. But most problems have alternative solutions that fulfill different goals and the phenotypes we observe are different trade-offs between them. Identifying the relevant goals of a certain process and the “design principles” relevant to achieve them enables a higher level of understanding and a much more efficient use of available data by building upon *a priori* knowledge.

In the case of metabolic pathways, modern methods have provided new ways for computers to do, what biochemists have always done. FBA is a way of following the traffic of molecules, just on a big scale. TFA applies the same principle to thermodynamics while BST/MCA add regulation to the picture. Applying these methods together within an integrative framework enables the analysis to be performed at the whole network level, rather than one pathway at a a time. This has been illustrated through small classical examples, like ammonia assimilation or a very simple pathway, but all the methods discussed have the potential to be used on a large scale. FBA is routinely performed on genome scale models and TFA is catching up fast, thanks to methods that approximate thermodynamic information for cases, where experimental data are not available [57]. The transition from constraint based to dynamic models is proving to be a difficult one, due to the non-linear nature of the mathematics involved but the use of standard formalisms, the connection between thermodynamics and sensitivity analysis, as well as the numeric extension of MCC [58], are highly promising strategies.

## Appendix

### A. Supplementary Information: The Unbranched Pathway

Compilation of previous results for the model used in case study 2. These results have been adapted from [3,55] and brought to a common notation consistent with that of this paper. A slightly more general representation of an unbranched pathway than the one in case study 2 would be:

The concentration of the first metabolite is assumed to be held constant and $x_{4}$ is a variable representing the demand for $x_{3}$, so $x_{0}$ and $x_{4}$ will be independent variables in the sense discussed in the introduction.

The reaction rates in power-law form can be written as, at least symbolically, from inspection of the pathway map. Every variable influencing a reaction, through mass-action or otherwise, will get a kinetic order and it will be positive or negative depending on whether the variable activates or inhibits the reaction. For instance, ${\text{v}}_{2}$ and ${\text{v}}_{3}$ will only depend on their substrates and products: ${\text{v}}_{i}=\alpha_{i}\;x_{i-1}^{g_{i,i-1}}\;x_{i}^{g_{i,i}}$. ${\text{v}}_{1}$ and ${\text{v}}_{4}$ will be assumed to be unaffected by their product concentrations, the first will have a (negative) kinetic order accounting for the end product inhibition ${\text{v}}_{1}=\alpha_{1}\;x_{0}^{g1,0}\;x_{3}^{g1,3}$ and the second will depend on the demand ${\text{v}}_{4}=\alpha_{4}\;x_{3}^{g4,3}\;x_{4}$. Now, we can write the dynamic model as a system of differential equations.

$$\begin{array}{cc} {\dot{x}}_{1}= & \;\alpha_{1}\;x_{0}^{g_{1,0}}\;x_{3}^{g_{1,3}}-\alpha_{2}\;x_{1}^{g_{2,1}}\;x_{2}^{g_{2,2}}\; \end{array}$$

(A1) $$\begin{array}{cc} {\dot{x}}_{2}= & \;\alpha_{2}\;x_{1}^{g_{2,1}}\;x_{2}^{g_{2,2}}-\alpha_{3}\;x_{2}^{g_{3,2}}\;x_{3}^{g_{3,3}}\; \end{array}$$

$$\begin{array}{cc} {\dot{x}}_{3}= & \;\alpha_{3}\;x_{2}^{g_{3,2}}\;x_{3}^{g_{3,3}}-\alpha_{4}\;x_{3}^{g_{3,4}}\;x_{4} \end{array}$$

The steady state equation for an S-system is:

(A2) $$A_{d}\;y_{d}=b-A_{i}\;y_{i}$$

where $A_{d}$ is a matrix formed with the kinetic orders and b is a vector formed with the rate constants.

When Equation (A2) is applied to our system, $y_{d}=\left(y_{1},y_{2},y_{3}\right)$, $y_{i}=\left(y_{0},y_{4}\right)$, the vector b has elements $b_{i}=\ln\alpha_{i+1}-\ln\alpha_{i}$ and the matrices are:

(A3) $$A_{d}=\left(\begin{array}{ccc} -g_{2,1} & -g_{2,2} & g_{1,3}\\ g_{2,1} & g_{2,2}-g_{3,2} & -g_{3,3}\\ 0 & g_{3,2} & g_{3,3}-g_{4,3} \end{array}\right)$$

(A4) $$A_{i}=\left(\begin{array}{cc} g_{1,0} & 0\\ 0 & 0\\ 0 & -1 \end{array}\right)$$

If the system was numerically defined, any necessary information could be obtained by inverting or multiplying a few matrices, but the linearity of the steady state also enables to work symbolically. For instance, using Cramer’s theorem, a symbolic solution can be obtained for the steady state. Let’s define matrix ${\left[A_{d}\right]}_{i}$ as the result of replacing column *i* in $A_{d}$ with the right hand side of Equation (A2). If we define Δ as the determinant of $A_{d}$, and $\Delta_{i}$ as the determinant of ${\left[A_{d}\right]}_{i}$,

(A5) $$y_{i}=\frac{\Delta_{i}}{\Delta }$$

which can have simple solutions, like in our example,

(A6) $$y_{3}\;=\;\frac{y_{4}+g_{1,0}\;y_{0}-b_{3}-b_{2}-b_{1}}{g_{4,3}-g_{1,3}}$$

but can also get extremely complicated as systems grow in size and complexity. Even in complex cases, it is often possible to obtain closed forms for the response of each flux to perturbations using careful determinant expansions. For instance, in the generalization of the unbranched pathway with *n* metabolites and an arbitrary pattern of feedback inhibitory loops, the response of end product to a change in precursor is [3]:

(A7) $$L_{x_{n},x_{0}}\;=\;\frac{\partial y_{n}}{\partial y_{0}}\;=\;\frac{{\left(-1\right)}^{n}\;\prod_{i=1}^{n}g_{i,i-1}}{\Delta }$$

where $L_{x_{i},x_{j}}$ is normally called the Logarithmic gain of $x_{i}$ with respect to $x_{j}$ and it is analogous to a control or response coefficient in MCA. Logarithmic gains are important for the analysis of the model because they measure the intensity of the response of our system to changes in its inputs or outputs. They loosely can be interpreted as a percent response. For instance, $L_{v,x_{4}}$ is the percent increase in flux that result in a 1% increase in demand.

In addition to sensitivity analysis, BST offers the possibility to perform stability analysis. The Routh criteria can be straightforwardly written in terms of: The kinetic orders, the steady state concentrations and the steady state flux. For the unbranched irreversible pathway with three intermediates used in the examples above, the stability criterion can be written as limit to the strength of the feedback inhibition (see [3] for details).

$$-g_{1,3}\;\;<\;\;g_{4,3}\;\left(2+\frac{F_{1}\;g_{2,1}}{F_{2}\;g_{3,2}}+\frac{F_{2}\;g_{3,2}}{F_{1}\;g_{2,1}}+\frac{F_{1}\;g_{2,1}}{F_{3}\;g_{4,3}}+\frac{F_{3}\;g_{4,3}}{F_{1}\;g_{2,1}}+\frac{F_{2}\;g_{3,2}}{F_{3}\;g_{4,3}}+\frac{F_{3}\;g_{4,3}}{F_{2}\;g_{3,2}}\right)$$

where $F_{i}=\frac{|v_{i}{|}_{0}}{|x_{i}{|}_{0}}$.

**Effect of reversible kinetics in the unbranched pathway** Alves and Savageau [47] analyzed the effect of reversible *vs.* fully irreversible kinetics in unbranched pathway models like Equation (25). Let’s define our reference system as a a fully irreversible version of Equation (25). In such case, $g_{2,2}=g_{3,3}=0$. The alternative system will differ in the second reaction, which, still being far away from equilibrium, is affected by its own product, $P≃K_{P}\Rightarrow {\hat{g}}_{2,2}\neq 0$. Since the characteristic difference between both systems lies in the second reaction, internal equivalence establishes that the parameters of all other reactions are equal: $\alpha_{i}={\hat{\alpha }}_{i}\;\;i\neq 2$ and $g_{i,j}={\hat{g}}_{i,j}\;\;i\neq 2$. External equivalence, on the other hand, helps us find values for the three parameters that are different in the alternative model: ${\hat{g}}_{2,1}$, ${\hat{g}}_{2,2}$ and ${\hat{\alpha }}_{2}$. By imposing that both pathways have the same concentrations of $x_{1}$, $x_{2}$, and $x_{3}$ in the steady state and that both have the same response to an increase in supply, it follows that

$$\begin{array}{cc} \log x_{i}\;= & \;\log\hat{x_{i}} \end{array}$$

(A8) $$\begin{array}{cc} L_{v,x_{0}}\;= & \;{\hat{L}}_{v,x_{0}}, \end{array}$$

Solving the equations above results in the following equivalence conditions:

(A9) $$\begin{array}{cc} {\hat{g}}_{2,1}\;= & \;\;\;\frac{g_{2,1}\;\left(g_{3,2}-{\hat{g}}_{2,2}\right)}{g_{3,2}} \end{array}$$

(A10) $$\begin{array}{cc} \log({\hat{\alpha }}_{2})\;= & \;\frac{g_{3,2}\;\log\left(\alpha_{2}\right)+g_{2,2}\;\left(\log\left(\alpha_{3}\right)-\log\left(\alpha_{2}\right)\right)}{g_{3,2}} \end{array}$$

As an additional consequence of the equivalence conditions, the response to demand of both systems is also the same $L_{v,x_{4}}={\hat{L}}_{v,x_{4}}$.

Once the two systems from the previous example have been made as similar as possible, they can be compared. Because the log-gains have been made equal, the differences between the systems will manifest through another set of sensitivities: the sensitivities to the rate constants. These give a good indication on how the system will respond to a change in the activity of each enzyme. The sensitivities of metabolite concentrations $S_{x_{i},\alpha_{j}}$ are also known as concentration control coefficients and the sensitivities of the fluxes $S_{v_{i},\alpha_{j}}$ are known as flux control coefficients. Most of this sensitivities are also identical in both systems, with the exception of:

S(x1,α2)=−1g2,1S^(x1,α2)=−g3,2g2,1(g3,2−g^2,2)S(x1,α3)=0S^(x1,α3)=−g^2,2g2,1(g3,2−g^2,2)

Since ${\hat{g}}_{2,2}<0$ and $\frac{g_{3,2}}{\left(g_{3,2}-{\hat{g}}_{2,2}\right)}<1$, the response of $x_{1}$ to $\alpha_{2}$ is always smaller (in absolute value) in the alternative system. The opposite happens with the response to $\alpha_{3}$, so we can see that making the second enzyme sensitive to product results in a transfer of control from the second to the third enzyme regarding the concentration of the first metabolite $x_{1}$. Everything else remains the same.

By combining symbolic and statistical methods, Alves and Savageau carried out a full analysis on the performance of an unbranched pathway with end-product inhibition depending on which reactions were fully irreversible ($g_{i,i}=0$) and which were reversible or sensitive to their product ($g_{i,i}\neq 0$) [55]. The results show that a pathway having all its reactions reversible is more stable and robust, than pathways having fully irreversible reactions in any of its steps. In general, they found that the most favorable position for a single fully irreversible step in an otherwise reversible pathway is the first and the best position for the only reversible step in a fully irreversible pathway is the last. A pathway with its first step irreversible and all the others reversible performs almost as well regarding stability as the fully reversible pathway but provides a much better response to demand: The response of the flux to an increase in demand is the highest while the decrease in end-product concentration is the smallest.

### B. Supplementary Information: Ammonia Assimilation

Conditions

All formation energies were taken from Equilibrator at pH = 7, I = 0.1 M, T = 298.15 K

**metabolites** glutamate 100 mM *α*-ketoglutaric acid 1 mM

**cofactors** [6] ATP = 7.9E-3 ADP = 1.04E-3 Pi = 7.9

Ratios [20]:

(A11) $$\begin{array}{cc} r_{N}\;= & \;\frac{\mathrm{NADP}}{\mathrm{NADPH}}=1.2 \end{array}$$

We can define NH_3_ as an independent variable $X_{0}$ and assume that the ratios of cofactors are fixed. The dependent variables will be *α* - ketoglutaric acid $X_{1}$, glutamine $X_{2}$ , glutamate $X_{3}$. We will also use the classical $y_{i}=\ln X_{i}$

**Glutamate dehydrogenase (GDH)**

NADPH + *α*–keto-glutarate + NH_3 <_ = > NADP^+^ + glutamate + H_2_O

(A12) $$\begin{array}{cc} \Delta G^{0^{\prime }}= & -38.9\;KJ/mol \end{array}$$

(A13) $$\begin{array}{cc} K_{eq}^{\prime }=\; & 6.6\cdot 10^{6} \end{array}$$

(A14) $$\Delta G^{{}^{\prime }}=\Delta G^{0^{\prime }}+RT\ln\frac{\left[{\mathrm{NADP}}^{+}\right]\;\left[\mathrm{glutamate}\right]}{\left[{\mathrm{NH}}_{3}\right]\;\left[\alpha -\mathrm{keto}-\mathrm{glutaric}\mathrm{acid}\right]\;\left[\mathrm{NADPH}\right]}$$

(A15) $$\Delta G^{{}^{\prime }}=\Delta G^{0^{\prime }}+RT\ln\frac{\left[{\mathrm{NADP}}^{+}\right]}{\left[\mathrm{NADPH}\right]}+RT\ln\frac{\left[\mathrm{glutamate}\right]}{\left[{\mathrm{NH}}_{3}\right]\;\left[\alpha -\mathrm{keto}-\mathrm{glutaric}\mathrm{acid}\right]}$$

(A16) $$\Delta G^{{}^{\prime }}=\Delta G^{0^{\prime }}+RT\ln r_{N}+RT\left(y_{3}-y_{0}-y_{1}\right)$$

**Glutamine synthase (GS)**

ATP + glutamate + NH_3 <_ = > ADP + glutamine + P_i_

(A17) $$\begin{array}{cc} \Delta G^{0^{\prime }}= & -15\;KJ/mol \end{array}$$

(A18) $$\begin{array}{cc} K_{eq}^{\prime }=\; & 423 \end{array}$$

(A19) $$\Delta G^{{}^{\prime }}=\Delta G^{0^{\prime }}+RT\ln\frac{\left[\mathrm{glutamine}\right]\;\left[\mathrm{ADP}\right]\;\left[P_{i}\right]}{\left[\mathrm{glutamate}\right]\;\left[{\mathrm{NH}}_{3}\right]\;\left[\mathrm{ATP}\right]}$$

(A20) $$\Delta G^{{}^{\prime }}=\Delta G^{0^{\prime }}+RT\ln\frac{\left[\mathrm{ADP}\right]\;\left[P_{i}\right]}{\left[\mathrm{ATP}\right]}+RT\ln\frac{\left[\mathrm{glutamine}\right]}{\left[\mathrm{glutamate}\right]\;\left[{\mathrm{NH}}_{3}\right]}$$

**Glutamate synthase (GOGAT)**

NADPH + *α* −keto-glutaric acid + glutamine _<_ = > NADP^+^ + 2 glutamate

(A21) $$\begin{array}{cc} \Delta G^{0^{\prime }}= & -50.3\;KJ/mol \end{array}$$

(A22) $$\begin{array}{cc} K_{eq}^{\prime }=\; & 6.7\cdot 10^{8} \end{array}$$

(A23) $$\Delta G^{\prime }=\Delta G^{0^{\prime }}+RT\ln\frac{{\left[\mathrm{glutamate}\right]}^{2}\;\left[{\mathrm{NADP}}^{+}\right]}{\left[\mathrm{NADPH}\right]\;\left[\alpha -\mathrm{keto}-\mathrm{glutaric}\mathrm{acid}\right]\;\left[\mathrm{glutamine}\right]\;}$$

**ATP hydrolisis**

ATP + H_2_O _<_ = > ADP + P_i_

(A24) $$\begin{array}{cc} \Delta G^{0^{\prime }}= & -26.4\;KJ/mol \end{array}$$

(A25) $$\begin{array}{cc} K_{eq}^{\prime }=\; & 4.2\cdot 10^{4} \end{array}$$

(A26) $$\Delta G^{\prime }=\Delta G^{0^{\prime }}+RT\ln\frac{\left[\mathrm{ADP}\right]\;\left[P_{i}\right]}{\left[\mathrm{ATP}\right]\;\left[H_{2}O\right]}$$
